# Functional Expression in *Escherichia coli* of the Disulfide-Rich Sea Anemone Peptide APETx2, a Potent Blocker of Acid-Sensing Ion Channel 3

**DOI:** 10.3390/md10071605

**Published:** 2012-07-23

**Authors:** Raveendra Anangi, Lachlan D. Rash, Mehdi Mobli, Glenn F. King

**Affiliations:** Institute for Molecular Bioscience, The University of Queensland, Brisbane, QLD 4072, Australia; Email: l.rash@uq.edu.au (L.D.R.); m.mobli@uq.edu.au (M.M.)

**Keywords:** ASIC3, APETx2, heterologous expression, NMR, *E. coli*

## Abstract

Acid-sensing ion channels (ASICs) are proton-gated sodium channels present in the central and peripheral nervous system of chordates. ASIC3 is highly expressed in sensory neurons and plays an important role in inflammatory and ischemic pain. Thus, specific inhibitors of ASIC3 have the potential to be developed as novel analgesics. APETx2, isolated from the sea anemone *Anthopleura elegantissima*, is the most potent and selective inhibitor of ASIC3-containing channels*.* However, the mechanism of action of APETx2 and the molecular basis for its interaction with ASIC3 is not known. In order to assist in characterizing the ASIC3-APETx2 interaction, we developed an efficient and cost-effective *Escherichia coli* periplasmic expression system for the production of APETx2. NMR studies on uniformly ^13^C/^15^N-labelled APETx2 produced in *E. coli* showed that the recombinant peptide adopts the native conformation. Recombinant APETx2 is equipotent with synthetic APETx2 at inhibiting ASIC3 channels expressed in *Xenopus* oocytes. Using this system we mutated Phe15 to Ala, which caused a profound loss of APETx2’s activity on ASIC3. These findings suggest that this expression system can be used to produce mutant versions of APETx2 in order to facilitate structure-activity relationship studies.

## 1. Introduction

Acid-sensing ion channels (ASICs) are voltage-independent, cation-selective channels that exist throughout the mammalian central and peripheral nervous systems. They are members of the degenerin/epithelial sodium channel (DEG/ENaC) protein superfamily and they play a critical role in sensing acidosis and mediating acid-induced cell injury [1]. Alternative splicing of four ASIC-encoding genes leads to the expression of six subunits: ASIC1a, ASIC1b, ASIC2a, ASIC2b, ASIC3, and ASIC4 [2]. Proton activation of ASICs is important in a variety of physiological and pathological conditions such as nociception, mechanosensation, synaptic plasticity, and acidosis-mediated neuronal injury [3]. Functional ASICs are formed as either homotrimeric or heterotrimeric channels. They are activated by external pH variations ranging from 7.0 to 4.0, and are characterized by either rapid (ASIC1a, ASIC1b, ASIC3) or slow (ASIC2a) kinetics of inactivation [4]. ASIC1a and ASIC3 are the subunits most sensitive to changes in pH (pH_0.5_ = 6.7). ASIC3 is predominantly distributed in the peripheral nervous system, namely in dorsal root ganglia (DRG) and trigeminal ganglia as well as in the sensory nerve endings of the skin and gastrointestinal tract [5,6,7]. A role for ASIC3 in response to heat, acid, mechanical stimuli, and as mediator of pain sensation during myocardial ischemia has been reported [7,8]. In an animal model studies, ASIC3 knockout mice are more sensitive to light touch and less sensitive to noxious pinches than normal mice, and they are resistant to the hyperalgesia induced by repeated intramuscular acid injections [6,9,10]. ASIC3 levels are elevated in inflamed knee joint afferent nerves and DRG neurons of rodents with inflamed hind paws, which suggests that increased levels of ASIC3 are related to an increased sensitivity to inflammatory stimuli [11,12,13,14]. More recent studies suggest that ASIC3 may also play broader ranging roles in non-pain related processes such as anxiety and insulin resistance (reviewed in [15]). 

The pharmacology of ASIC3 is still relatively poorly understood when compared with voltage-gated sodium (Na_V_) and calcium (Ca_V_) channels [16]. Thus, the characterization of, and development of structure-activity relationships for, selective ASIC3 modulators will be useful for characterizing the function of ASIC3 under both normal and pathophysiological conditions. With the exception of protons, 2-guanidine-4-methylquinazoline (GMQ) is the only other known ASIC3 agonist, causing persistent activation at normal pH [17]. The majority of currently known inhibitors of ASIC3 (e.g., gadolinium, amiloride, and some non-steroidal anti-inflammatory drugs) are neither potent nor specific [18]. The exception to this is APETx2, a specific peptide blocker of ASIC3 isolated from the sea anemone *Anthopleura elegantissima* [19]. APETx2, also known as π-AITX-Ael2b using the recently introduced rational nomenclature for sea anemome toxins [20], is a 42-residue peptide that inhibits ASIC3-containing channels (ASIC3, ASIC3/1a, ASIC3/1b, ASIC3/2b) with an IC_50_ ranging from ~60 nM for homomeric ASIC3 up to 2 μM depending up on the subunit composition of the channel [19]. APETx2 has subsequently been shown to be analgesic in rodent pain models [21,22] and the peptide is in preclinical studies as a potential analgesic [23]. A recent study revealed that APETx2 also inhibits Na_V_ 1.8, another novel pain target, which may contribute to its analgesic activity [24]. To date very little is known about the mechanism of action or active surface of APETx2. This is largely due to limited access to sufficient amounts of material.

The major disadvantages of isolating APETx2, or indeed any venom peptide, from natural sources are potentially limited access to animals and the need for multi-step purifications (due to the complex nature of the starting material), which usually results in low yields [25]. Therefore, production of APETx2 by other routes is required to obtain adequate material for structure-function studies. Previously, APETx2 has been synthesized by solid-phase peptide synthesis and native chemical ligation methods (SPPS/NCL) [26]. This method is relatively efficient but requires several purification steps, oxidative refolding of the peptide, and further purification. Furthermore, incorporation of stable isotopes (^15^N and/or ^13^C) for NMR studies using this approach is prohibitively expensive. Here we report the use of an *E. coli* periplasmic expression system for the expression of soluble, correctly folded APETx2. Using this recombinant method we were able to produce both unlabelled and uniformly labelled (^15^N/^13^C) APETx2 in soluble form and demonstrate its utility for producing directed mutations to aid structure-activity relationship (SAR) studies. 

## 2. Results and Discussion

### 2.1. Production of Recombinant APETx2 and Its F15A Mutant

**Figure 1:**
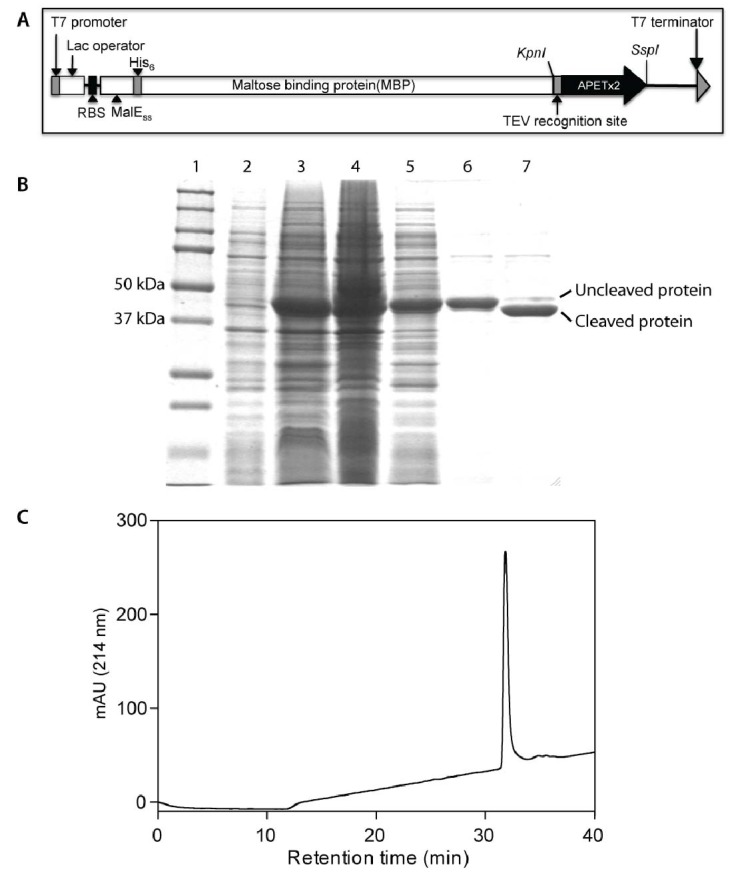
(**A**) Schematic of construct encoding a His_6_-MBP: APETx2 fusion protein for production of rAPETx2 in *E. coli*. (**B**) SDS-PAGE gel showing expression and purification of rAPETx2. Lane 1: molecular mass standards (the size of selected standards is shown on left of gel); Lanes 2 and 3: *E. coli* cells before and after induction with IPTG; Lanes 4 and 5: insoluble and soluble fractions resulting from rupture of *E. coli* cells; Lanes 6 and 7: purified MBP fusion protein before and after cleavage with TEV protease. (**C**) RP-HPLC chromatogram showing the final purification of folded rAPETx2.

Recombinant production of sea anemone peptides is challenging as they possess multiple disulfide bonds. The cytoplasm of *E. coli* is a reducing environment and not favourable for the production of cysteine-rich venom peptides [27]. In order to avoid this problem it is possible to direct an expressed protein to the *E. coli* periplasm, where oxidative folding of proteins takes place with the help of the Dsb family of proteins [27]. We previously demonstrated the applicability of this approach for production of π-TRTX-Pc1a, a disulfide-rich spider-venom peptide that specifically inhibits ASIC1a [28], and a similar approach was used to produce the spider-venom peptide huwentoxin-I [29]. π-TRTX-Pc1a is relatively hydrophilic and easy to fold *in vitro*, while APETx2 is substantially more hydrophobic and difficult to fold, thus posing a greater challenge for recombinant production. The plasmid construct used to encode an IPTG-inducible MBP-APETx2 fusion protein is shown in Figure 1A. In addition to the codon-optimized APETx2 gene, it consists of a MalE signal sequence (to direct the fusion protein to the periplasm), an *N*-terminal His_6_ tag for affinity purification, a maltose binding protein (MBP) tag to aid solubility, and a tobacco etch virus (TEV) protease cleavage site between the MBP and APETx2 coding regions.

Using this vector, APETx2 was the major cellular protein produced after IPTG induction of transformed *E. coli* BL21(λDE3) cells (compare lanes 2 and 3 in Figure 1B). After cell rupture, ~50% of the MBP: APETx2 fusion protein was recovered in the soluble cell fraction (compare lanes 4 and 5 in Figure 1B). The fusion protein was purified from the soluble cell fraction using nickel affinity chromatography, resulting in a partially purified protein of ~46 kDa (Figure 1B, lane 6). More than 90% of the fusion protein was cleaved to yield free rAPETx2 following incubation with TEV protease (Figure 1B, lane 7). After TEV cleavage, rAPETx2 was purified to ~98% homogeneity using reversed-phase high performance liquid chromatography (RP-HPLC; Figure 1C). The *E. coli* periplasmic expression system resulted in a yield of approximately 1.0 mg/L of soluble rAPETx2. 

**Figure 2:**
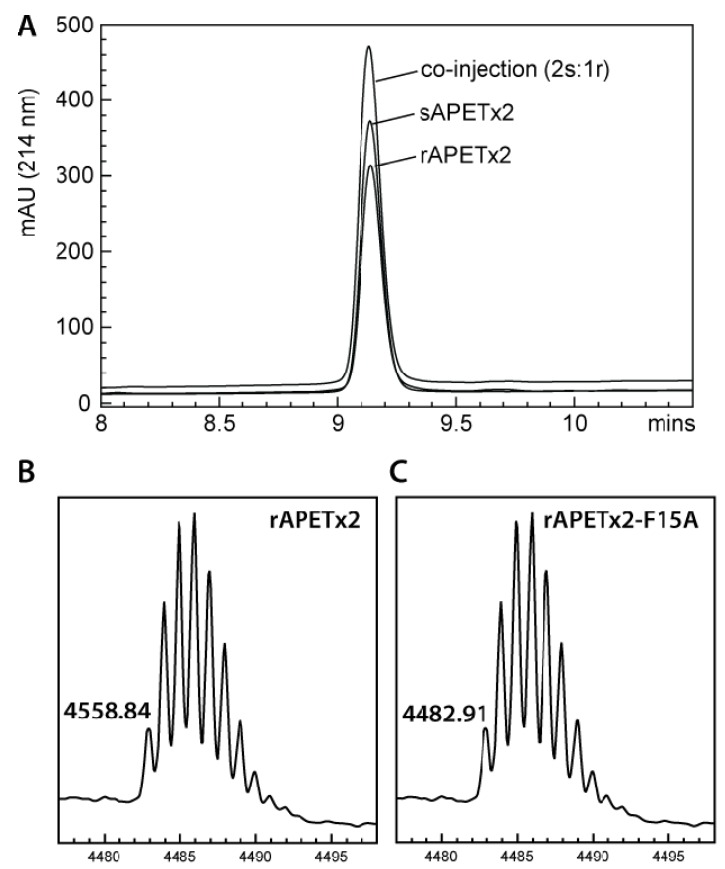
(**A**) RP-HPLC chromatogram showing co-elution of synthetic and recombinant APETx2. (**B**) MALDI-TOF mass spectrum showing *m/z* of pure rAPETx2. (**C**) MALDI-TOF mass spectrum showing *m/z* of pure rAPETx2-F15A.

Analysis of purified rAPETx2 using MALDI-TOF mass spectrometry indicated that rAPETx2 produced in *E. coli* periplasm is fully oxidized, with three-disulfide bonds. The observed monoisotopic mass (M + H^+^) of rAPETx2 was 4558.8 (Figure 2B), which is in good agreement with the calculated monoisotopic mass of oxidized APETx2 (4558.9) but not the reduced peptide (4564.9). With three disulfide bonds, rAPETx2 can potentially form 15 different disulfide-bond isoforms and therefore it is important to confirm that the native disulfide-bond isomer has been obtained regardless of the method of peptide production. To this end, we showed that rAPETx2 co-eluted on RP-HPLC with synthetic APETx2 that we previously demonstrated has identical activity to APETx2 from the native source [26]; when the synthetic and recombinant peptides were co-injected in a 2:1 ratio, a symmetrical peak was obtained showing they are indeed the same isoform (Figure 2A).

**Figure 3:**
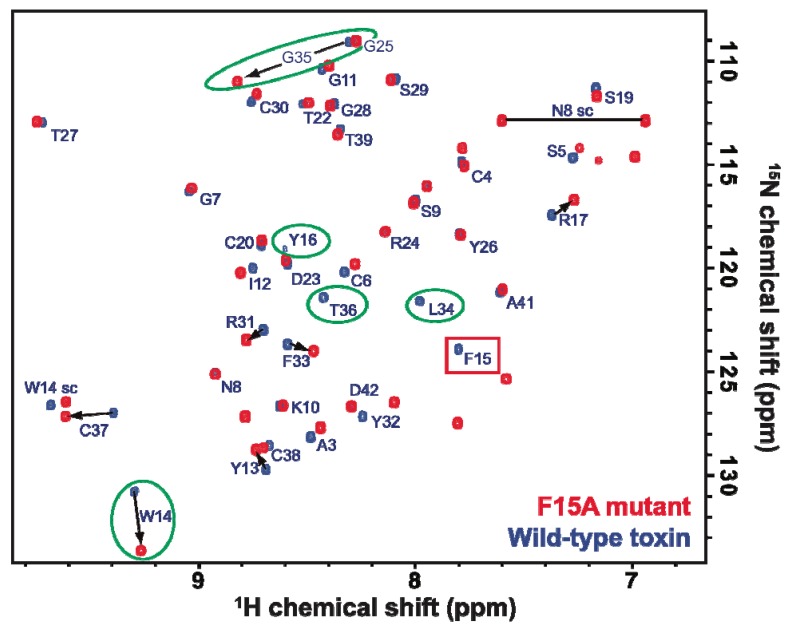
Overlaid 2D ^1^H-^15^N HSQC spectrum of uniformly ^15^N-labelled wild-type rAPETx2 (blue) and a F15A mutant (red). Residues whose chemical shifts are most affected by the mutation of Phe15 to Ala are highlighted with green circles while the peak corresponding to F15 in the wild-type toxin is shown in a red box. The horizontal line connects the two peaks from the sidechain amide group of Asn8, and “W14 sc” indicates the peak from the sidechain indole NH group of Trp14.

In addition to simplicity and low cost, the *E. coli* expression system has the distinct advantage of allowing economical incorporation of isotopic labels, such as ^2^H, ^13^C, and ^15^N typically used for NMR studies. A fully assigned 2D ^1^H-^15^N HSQC spectrum of uniformly ^15^N-labelled rAPETx2 produced using the periplasmic expression system described here is shown in Figure 3. The excellent chemical shift dispersion in both the ^1^H and ^15^N frequency dimensions indicates that the peptide is folded into ordered tertiary structure and the chemical shifts are very similar to those reported previously for rAPETx2 produced using *Pichia pastoris* [30].

### 2.2. Activity of Recombinant APETx2 and Its F15A Mutant

**Figure 4:**
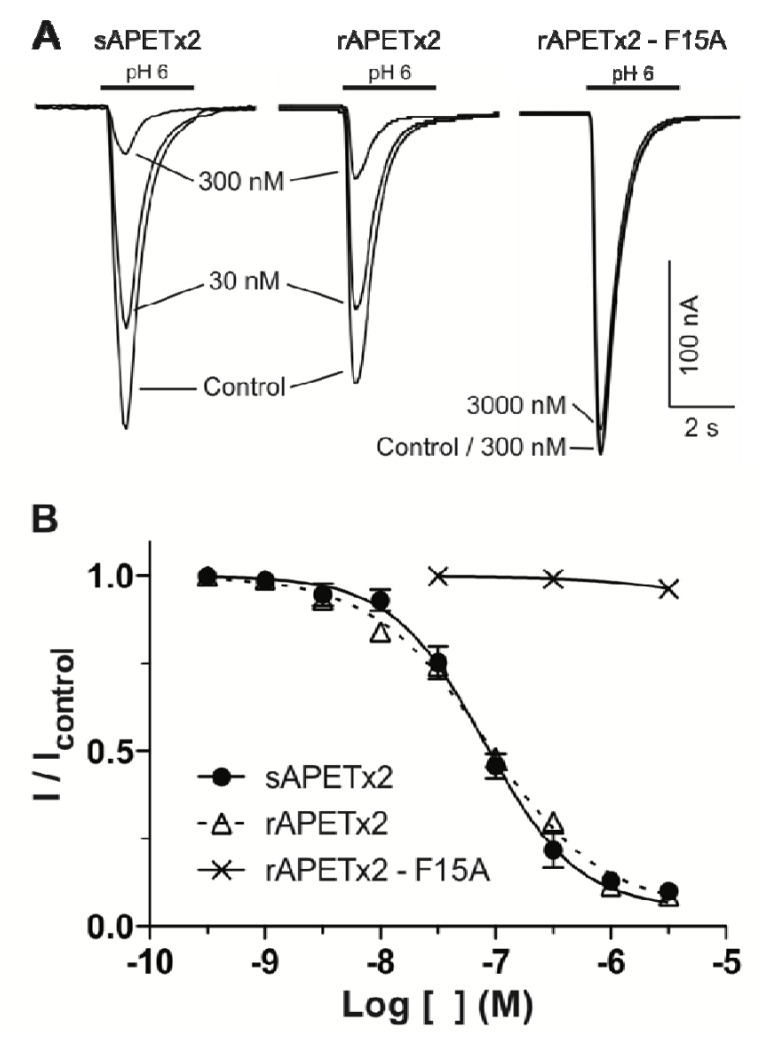
(**A**) Representative current traces comparing the concentration-dependent inhibition of rat ASIC3 (expressed in *Xenopus* oocytes) by synthetic (s) and recombinant (r) APETx2. (**B**) Concentration-effect curves for sAPETx2 and an rAPETx2–F15A mutant.

The efficacy of rAPETx2 was tested on rat ASIC3 expressed in *Xenopus laevis* oocytes. Nanomolar concentrations of rAPETx2 caused a concentration-dependent inhibition of homomeric ASIC3 channels stimulated every minute by a pH drop from 7.4 to 6.0 (Figure 4A). Using this system, rAPETx2 was found to be equipotent with sAPETx2; the synthetic and recombinant peptide both had an IC_50_ of 80 nM (Figure 4B), which corresponds well with the value of 63 nM reported for native APETx2 [19] and synthetic APETx2 from several sources [24,26]. Thus, rAPETx2 produced in the periplasm of *E. coli* is equipotent with native toxin. 

Little is known about which residues on APETx2 are important for its inhibition of ASIC3. However, when determining the solution structure of APETx2, Chagot and colleagues suggested a possible binding surface based on the orientation of the dipole moment that results from its electrostatic anisotropy [31]. This putative functional surface is made up of a basic/aromatic cluster of residues in loops two and four of the peptide, including Phe15. We decided to test this hypothesis and further demonstrate the utility of the *E. coli* periplasmic expression system by producing a F15A mutant. The mutant was obtained in similar yield to the native peptide, and mass spectrometry (Figure 2C) and NMR HSQC data (Figure 3) obtained for the F15A mutant revealed that it was fully oxidized and adopted the native fold. As shown in Figure 3, the large majority of peaks in the HSQC spectrum of APETx2 are unaffected by the F15A mutation. Significant peak shifts were observed for very few residues, and all of these can be attributed to a loss of the ring current shift due to removal of the aromatic ring of F15. The affected residues include the neighboring residues W14 and Y16 and residues 34–36 (all circled in green in Figure 3). The latter residues lie at the tip of a β-strand immediately adjacent to F15 on the neighbouring β-strand, and they are well within the distance over which F15 would be expected to exert a ring current shift effect. We therefore conclude that the F15A mutation does not significantly perturb the tertiary structure of APETx2. However, electrophysiological measurements revealed that changing F15 to alanine reduced the ability of APETx2 to inhibit ASIC3 by more than 100-fold (Figure 4). These data show that Phe15 is extremely important for APETx2 inhibition of ASIC3 activity.

### 2.3. Discussion

Although the physiological roles of ASIC3 are not fully understood, there is growing evidence to suggest that it has many physiological and pathological functions. Recent studies have revealed that ASIC3 is localized in several specialized sensory nerve endings of skin, muscle, arteries, inner ear, visceral tissues and in non-neuronal tissues such as bone, lung and testis [15,32]. One of the most intensively studied aspects of ASIC function is in the context of inflammatory and ischemic pain [8,9,13,14,32,33,34]. Therefore, it is not surprising that ASIC3 has emerged as a potential drug target for developing novel analgesics [1,15].

APETx2 is the only known potent and selective inhibitor of ASIC3 [19]. It also inhibits Na_V_1.8, another analgesic target, albeit with lower potency than for ASIC3 [24]. APETx2 has been shown to abolish acid-induced and post-operative pain in rats [21,22,35] and the peptide is in preclinical studies as a potential analgesic [23]. Establishment of an efficient and cost-effective method of APETx2 production should expedite future SAR studies on this peptide, which in turn will facilitate rational engineering of more potent and selective analogues.

We previously produced APETx2 using a combination of solid-phase peptide synthesis and native chemical ligation, which necessitates oxidative refolding the peptide [26]. Although this is relatively efficient and allows the incorporation of non-natural amino acids and modifications, chemical synthesis of cysteine-rich peptides can be expensive, especially if one wishes to incorporate stable isotopes such as ^15^N and ^13^C in order to increase the speed and resolution obtainable from NMR structural studies [36]. Recombinant expression is broadly considered the most cost-effective approach for production of both unlabelled and uniformly-labelled cysteine-rich peptides. Recombinant expression can be carried out using either prokaryotic or eukaryotic systems, but there are very few examples of production of disulfide-rich peptides in eukaryotic systems and the yields reported have been low; for example, the spider-venom peptide π-TRTX-Pc1a was successfully produced in an insect cell line but with a relatively low yield of 0.5 mg/L [37].

Recently, a yeast expression system was developed to produce recombinant APETx2, which was secreted into the growth medium [30]. Although the rAPETx2 produced in *P. pastoris* was fully functional, yeast take considerably longer to grow than *E. coli* and the rAPETx2 produced in *P. pastoris* required an arduous three-step purification in order to obtain the correct disulfide-bond isomer [30]. Furthermore, the incorporation of isotopic labels into peptides in *P. pastoris* is very expensive. The most common host for heterologous expression of venom peptides is *E. coli* but this often necessitates the oxidative refolding of the peptides as the redox environment of the *E. coli* cytoplasm does not favour formation of disulfide bonds [27,38,39]. Recently we developed an *E. coli* periplasmic expression system for production of disulfide-rich venom peptides [25,28]. Higher yields of correctly folded peptide can often be obtained by exporting proteins into the periplasm because this is where the cellular machinery for disulfide-bond formation is located in *E. coli* [25,28]. In the current study, we successfully adapted this *E. coli* periplasmic expression system for efficient production of rAPETx2, which we demonstrated to be equipotent with the native toxin. The final yields obtained for APETx2 produced in *P. pastoris* were 2–4 mg/L and 0.5–1.0 mg/L for unlabelled and ^15^N labelled peptides, respectively [30]. The final yields obtained using the *E. coli* periplasmic expression system are slightly lower (~1.0 mg/L and ~0.5 mg/L for unlabelled and ^15^N labelled toxin, respectively). However, the recombinant APETx2 obtained using *P. pastoris* expression as reported by Anangi *et al.* [30] contained an *N*-terminal His_6_ tag and additional residues (EF) from mis-cleavage of the signal sequence, whereas the rAPETx2 reported here has no vestigial residues or tags and thus represents the native sequence. Considering this fact, as well as the reduction in time required for expression and purification as well as the cost of isotopically enriched medium, the *E. coli* periplasmic expression seems to be a time and cost effective option for production of APETx2. Moreover, *E. coli* expression enables much easier and cheaper incorporation of ^13^C labels for NMR studies than expression in *P. pastoris*.

**Figure 5:**
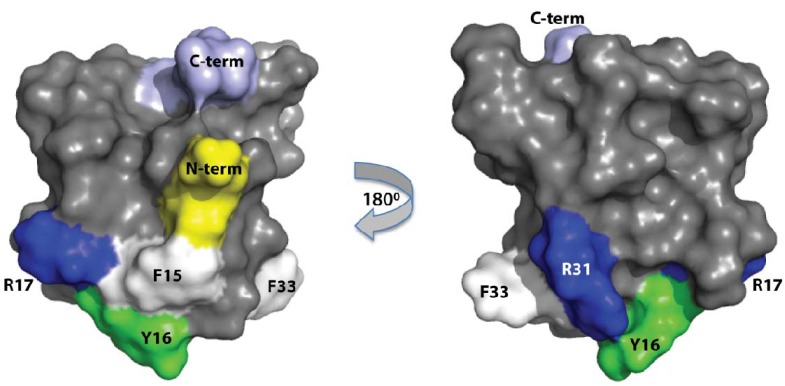
Three-dimensional solution structure of APETx2 (PDB accession code 1WXN) showing the relative positions of the *N*- and *C*-termini, and the predicted interaction surface including the confirmed pharmacophore residues R17 [30] and F15 (this study).

Based on the dipole moment calculated from the NMR solution structure of APETx2, Chagot and colleagues proposed that a cluster of positively charged and hydrophobic amino acids (F15, Y16, R17, R31 and F33) is likely to be the primary surface that mediates the toxin’s interaction with ASIC3 [31]. This prediction was supported by a recent study showing that mutation of R17 to Ala causes a 25-fold decrease in the ability of APETx2 to inhibit mouse ASIC3 [30]. In the current study, we further tested the prediction of Chagot and colleagues by making an F15A mutant. Mutation of F15 to Ala in the second loop of APETx2 reduced the toxin’s potency on ASIC3 by more than 100-fold. This suggests that F15 is a key residue for the efficient interaction of APETx2 with ASIC3. The structure of APETx2 (Figure 5) shows that F15 and R17 are spatially proximal and close to other residues predicted by Chagot and colleagues to be important for the interaction with ASIC3. Thus, it appears that, as shown recently for π-TRTX-Pc1a [28], a patch of aromatic and positively charged amino acids on the surface APETx2 (shown in Figure 5) is likely to mediate the toxin’s interaction with ASIC3.

## 3. Experimental Section

### 3.1. Production and Purification of Recombinant APETx2

Synthetic genes encoding APETx2 or APETx2-F15A, with codons optimized for expression in *Escherichia coli*, were cloned into a variant of the pLic-MBP expression vector [28,40]. This vector encodes a MalE signal sequence for periplasmic export, a His_6_ affinity tag, a maltose binding protein (MBP) fusion tag (to aid solubility), and a TEV protease recognition site directly preceding the APETx2-coding region (Figure 1A). Plasmids were transformed into *E. coli* strain BL21(λDE3) for recombinant toxin production.

Cultures were grown in Luria-Bertani medium at 37 °C with shaking at 180 rpm. Expression of the toxin gene was induced with 1 mM IPTG at an OD_600_ of 0.8 to 1.0 and cells were harvested 3 h later by centrifugation for 10 min at 6000 rpm. For production of uniformly ^13^C/^15^N-labelled APETx2, cultures were grown in minimal medium supplemented with ^13^C_6_-glucose and ^15^NH_4_Cl as the sole carbon and nitrogen sources, respectively. Two approaches were initially trialed for recovering the His_6_-MBP-toxin fusion protein: Cells were lysed using a high-pressure cell disruptor (TS Series Benchtop model, Constant Systems Ltd., UK) or the fusion protein was extracted from the bacterial periplasm by osmotic shock using 30 mM Tris, 40% sucrose, 2 mM EDTA pH 8.0 and ice-cold water. The yields of fusion protein were similar using both approaches and therefore whole-cell lysis was used in all subsequent experiments as it is faster and simpler. After cell lysis, soluble His_6_-MBP-toxin fusion protein was captured by passing the soluble cell extract (buffered in 20 mM Tris, 200 mM NaCl, 10% glycerol, pH 8.0) over an Ni-NTA Superflow resin (QIAGEN, Valencia, CA, USA) followed by washing with 25 mM imidazole to remove nonspecific binders. The fusion protein was then eluted with 300 mM imidazole. The buffer was exchanged to remove imidazole, then reduced and oxidized glutathione were added to 3 and 0.3 mM, respectively, to activate TEV protease and promote folding of the protein. Approximately 40 μg of His_6_-tagged TEV protease was added per mg of APETx2, and the cleavage reaction was allowed to proceed at room temperature for 12 h. The cleaved His_6_-MBP and His_6_-TEV were removed by passing the solution over Ni-NTA Superflow resin, whereas the eluate containing cleaved APETx2 was collected for further purification using RP-HPLC. RP-HPLC was performed on a Vydac C18 column (250 × 4.6 mm; particle size, 5 μm) using a flow rate of 1 mL/min and a gradient of 20%–40% solvent B (0.1% trifluoroacetic acid (TFA) in 90% acetonitrile) in solvent A (0.1% TFA in water) over 40 min. 

### 3.2. MALDI-TOF Mass Spectrometry

Toxin masses were confirmed by matrix-assisted laser desorption ionization–time-of-flight mass spectrometry (MALDI-TOF MS) using a model 4700 Proteomics Bioanalyser (Applied Biosystems, Foster City, CA, USA). RP-HPLC fractions were mixed [1:1 (v/v)] with α-cyano-4-hydroxy-cinnamic acid matrix (5 mg/mL in 50/50 acetonitrile/H_2_O, 0.1% TFA) and MALDI-TOF mass spectra were collected in positive reflector mode. All masses given are for the monoisotopic M + H^+^ ions.

### 3.3. NMR Spectroscopy

Lyophilized recombinant APETx2 was reconstituted at a final concentration of 300 μM in 10 mM sodium phosphate buffer, pH 6.0, constituted in 90% H_2_O/10% D_2_O. The sample was filtered using a low-protein binding Ultrafree-MC centrifugal filter (0.22-μm pore size; Millipore, Billerica, MA, USA), then 300 μL was added to a susceptibility-matched 5-mm outer diameter microtube (Shigemi Inc., Japan). Data were acquired at 298 K using a Bruker Avance III 900 MHz spectrometer equipped with a cryogenically cooled probe (Bruker BioSpin GmbH, Rheinstetten, Germany). 

### 3.4. Electrophysiological Measurements

Oocytes were obtained from *Xenopus laevis* and defolliculatd by treatment with collagenase (Sigma type I). Rat ASIC3 cRNA was synthesised using an mMessage mMachine cRNA transcription kit and healthy stage V–VI oocytes injected with 4 ng rat ASIC3 cRNA (40 nL of 100 ng/μL). Oocytes were kept at 17 °C in ND96 solution (96 mM NaCl, 2 mM KCl, 1 mM CaCl_2_, 2 mM MgCl_2_, 5 mM HEPES, 5 mM pyruvic acid, 50 µg/mL gentamicin, 2.5% fetal horse serum, pH 7.4). Membrane currents were recorded 2–6 days post cRNA injection under voltage-clamp (Axoclamp 900A amplifier, Molecular Devices, Sunnyvale, CA, USA) using two standard glass microelectrodes with resistances of 0.5–1 MΩ when filled with 3 M KCl solution. Data acquisition and analysis were performed using pCLAMP software (Version 10, Molecular Devices, Sunnyvale, CA, USA). Oocytes were clamped at −60 mV with data sampled at 1000 Hz and filtered at 0.01 Hz. All experiments were performed at RT (18–21 °C) in ND96 solution containing 0.1% fatty acid free-bovine serum albumin (BSA). Changes in extracellular pH were induced using a microperfusion system that allowed local and rapid changes of solutions. HEPES was replaced by MES to buffer the pH 6 stimulus solution. Toxin stock solutions were made up in ND96, 0.1% BSA (pH 7.4) to either 1 or 3 μM and serial dilutions were made from these in ND96 solution containing 0.1% BSA to prevent adsorption to tubing.

## 4. Conclusions

Numerous venom-derived disulfide-rich peptides are currently in preclinical and clinical development as potential therapeutics [41]. A potential roadblock in furthering many of these studies is the lack of an efficient method for toxin production. Here we have shown that the sea anemone peptide APETx2, which is currently in preclinical development as a potential analgesic, can be efficiently produced by expression in the periplasm of *E. coli*. This system allows facile production of native toxin as well as mutants and isotopically-labelled peptide for NMR structural studies. The approach described here should facilitate future SAR studies of not only APETx2 and related sea anemone peptides but also other disulfide-rich venom peptides being developed as drug or insecticide leads. 
